# Author's Reply

**DOI:** 10.1111/vox.12798

**Published:** 2019-06-17

**Authors:** Hans Van Remoortel, Emmy De Buck

**Affiliations:** ^1^ Centre for Evidence‐Based Practice Belgian Red Cross Mechelen Belgium; ^2^ Department of Public Health and Primary Care, Faculty of Medicine KU Leuven Leuven Belgium

We would like to thank T‐H Kim and JW Kang for carefully reading our paper. The authors state in the title of their letter that there could not be a strong association between acupuncture and HCV infection, a statement that should not be made neither based on our systematic review (SR), where we found a significant association (OR 1·56, 95%CI [1·17, 2·08]) based on very‐low‐quality evidence, nor on the additional data they provide. We would like to explain this in more detail and further comment on the methodological ‘issues’ that were raised by the authors.

Firstly, the authors refer to one single study 1 to state that there is no strong or an uncertain association between acupuncture and HCV infection. Formulating a conclusion by cherry‐picking one study is at odds with conducting a rigorous SR. This study was not included in our review since it was not conducted in blood donors. In addition, the conclusion the authors attribute to this study, in this and also in a previously published letter 2, is too strong. The study reports an OR of 1·06, 95%CI [0·73, 1·53]; however, this does not equal ‘evidence of no effect’ (i.e. ‘acupuncture was reported to be irrelevant to HCV infection’), but rather ‘no evidence of effect’ (i.e. ‘an association between acupuncture and HCV infection cannot be demonstrated’). The reason for this is that there is imprecision (i.e. large variability in results/large confidence interval) according to the GRADE methodology 3. According to GRADE, this study also contains very‐low‐certainty evidence, similarly to our SR, which means that it is uncertain whether the research provides a reliable indication of the true effect.

Secondly, the authors state that we omitted two studies from the meta‐analysis. However, a lack of data was present in 1 study since no information on the controls was reported 4. The other study only reported a univariate (significant) OR, but did not report the non‐significant multivariate OR, and therefore, we excluded this study from the meta‐analysis 5. When including this study, we then see that the significant association between acupuncture and HCV infection even increases, as this study, showing a significant association between acupuncture and HCV infection in itself 5, has a weight of 32% to the total pooled value.

Lastly, the authors asked to consider risk differences (RD) as effect measure to avoid problems with zero‐event studies when calculating the OR. The resulting forest plot (with the additional study included) can be found with this letter, showing that the significant OR now changed into a non‐significant RD of 0.06, 95%CI [−0·02, 0·14]. However, according to the Cochrane manual, RD methods yield too wide confidence intervals when events are rare, which make them unsuitable for meta‐analysis of rare events 3. We will therefore not rely on these results.

In summary, the available evidence does not support the conclusion that there could not be a strong association between acupuncture and transfusion‐transmissible infections. In order to gain more insight in this association, better quality studies are necessary.

**Figure 1:**
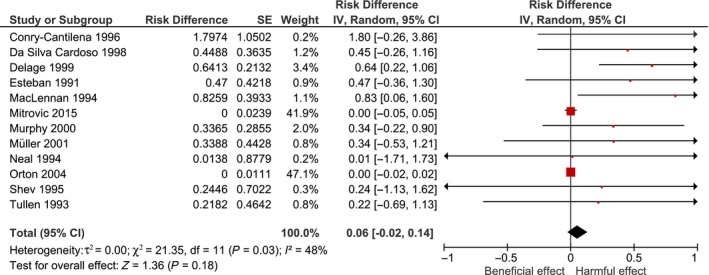
Study‐specific risk differences (RDs) representing the association between acupuncture and HCV infections in blood donors. [Colour figure can be viewed at http://wileyonlinelibrary.com]
